# Sampling efficiency and screening of *Aedes albopictus* for yellow fever virus in Niger Delta region of Nigeria

**DOI:** 10.11604/pamj.2024.47.120.39462

**Published:** 2024-03-14

**Authors:** Chioma Cynthia Ojianwuna, Victor Ngozi Enwemiwe, Andy Ogochukwu Egwunyenga, Akwilla Agboro, Emmanuel Owobu

**Affiliations:** 1 Department of Animal and Environmental Biology, Faculty of Science, Delta State University, Abraka, Nigeria

**Keywords:** *Aedes albopictus*, Delta State, Ika North East, molecular screening, sampling efficiency

## Abstract

**Introduction:**

Aedes albopictus, like Aedes aegypti, is a virulent vector of arboviruses especially the well-documented spread of yellow fever around the world. Although yellow fever is prevalent in Nigeria, there is a paucity of information in the Niger Delta region on the distribution of Aedes mosquito vectors and molecular detection of the virus in infected mosquitoes. This study sampled Aedes mosquitoes around houses associated with farms from four communities (Otolokpo, Ute-Okpu, Umunede, and Ute Alohen) in Ika North-East Local Government Area of Delta State, Nigeria.

**Methods:**

various sampling methods were used in Aedes mosquito collection to test their efficacy in the survey. Mosquitoes in holding cages were killed by freezing and morphologically identified. A pool of 15 mosquitoes per Eppendorf tube was preserved in RNAi later for yellow fever virus screening. Two samples were molecularly screened for each location.

**Results:**

seven hundred and twenty-five (725) mosquitoes were obtained from the various traps. The mean abundance of the mosquitoes was highest in m-HLC (42.9) compared to the mosquitoes sampled using other techniques (p<0.0001). The mean abundance of mosquitoes was lowest in Center for Disease Control (CDC) light traps without attractant (0.29). No yellow fever virus strain was detected in all the mosquitoes sampled at the four locations.

**Conclusion:**

this study suggests that Aedes albopictus are the mosquitoes commonly biting around houses associated with farms. More so, yellow fever virus was not detected in the mosquitoes probably due to the mass vaccination exercise that was carried out the previous year in the study area. More studies are required using the m-HLC to determine the infection rate in this endemic area.

## Introduction

The Asian tiger mosquito, *Aedes albopictus* (Skuse, 1894), like other species of *Aedes* is a widespread vector of several arboviruses affecting humans and animals in tropical and subtropical regions [1]. They bite their host to transmit the viruses that cause Dengue fever virus, Japanese encephalitis, Zika virus, Chikungunya, yellow fever viruses, and many others [2,3]. Globally, up to 4000 mosquito species have been reported in 41 genera [4], but only three genera including *Anopheles, Aedes*, and *Culex* are known as efficient vectors of these diseases in Africa [5]. The other species of *Aedes* include *Aedes aegypti* (Linnaeus in Hasselquist, 1762), *Ae. africanus* (Theobald, 1901), *Ae. Pseudoscutellaris* (Theobald, 1901), *Ae. Malaysia* (Colless, 1963), *Ae. Alcasidi* (Huang, 1972), *Ae. polynesiensis* (Marks, 1951) and many others are responsible for the viral diseases ravaging endemic areas in sub-Saharan Africa and other parts of the world [6]. In Nigeria, Dengue fever virus, Chikungunya, malaria, West Nile virus, lymphatic filariasis, Rift Valley fever virus, and yellow fever have been reported in patients visiting health facilities [7]. The vector utilizes stagnant water in containers, tires, cellophane in dumpsites, and several habitats for breeding. A study has confirmed that unvaccinated individuals who visit or live in endemic areas acquire the infection leading to an alarming increase in the number of yellow fever virus (YFV) cases and deaths [8]. Importantly, farmers who cultivate in areas where *Aedes* are abundant may have the likelihood of being infected [9].

Yellow fever virus (YFV) is one of the human flaviviral infections of the family Flaviviridae. In addition to humans being the primary host, forest animals can serve as reservoir hosts that sustain the infection pool. A historic account by Bryant JE *et al*. and Huang YLS *et al*. have reported the occurrence of YFV in Africa and the advertent spread to other regions of the world with the slave trade as far back as the mid-16^th^ century [10,11]. The presence of YFV infection in the blood of slaves could trigger infections in new areas where the vectors are abundant. Since the American slave masters lived with the slaves, the chances of transmission were high. In the mid-16^th^ century, between the 1640s and 1650, a YFV outbreak occurred and another occurred in the late 16^th^ century. Today, the viral infection is endemic in up to 50 African countries, Southern and Central America where they cause high morbidities and mortalities [3]. In 2013, a severe infection of YFV was estimated in about 200,000 persons and death reached 60,000 [3]. This infection is present in Nigeria, whereby 1,312 persons in 367 local government areas (LGAs) are suspected carriers [3]. Positive cases of YFV have been confirmed only by screening blood samples, and it is confirmed to be high in nine states such as Ondo, Delta, Benue, Niger, Enugu, Anambra, Oyo, Benue, and Osun states are affected [3,12].

The Nigerian population is highly vulnerable to YFV infection and intervention targeting the infection is by vaccination. The expected World Health Organization (WHO) target for YFV vaccination was 80% but there was a decline in the coverage between 2018 and 2020 which was reportedly 54% [3]. However, the mass vaccination done between 2019 and 2020 showed that Delta and Ondo's states had over 90% coverage compared to other states. The first outbreak of YFV in Delta State was reported in 2019 with one case in Ika North East, but an increased outbreak has been reported in Enugu State, both in Southern Nigeria [3].

The global mandate to Elimination of yellow Fever Epidemics (EYE) has targeted vectors and human hosts to monitor the infection rate. Humans are protected from YFV by getting vaccinated [13] and using insecticides [14]. Insecticides can be introduced into Long Lasting Insecticide Treated Nets (LLINs), used as indoor residual sprays (IRS), other appropriate treated materials, and insecticide larviciding [15]. Biolarviciding is equally common using *Bacteria thuringiensis* (BTi) and insect growth regulators (IGR). The excessive use of insecticides has led to the issues of insecticide resistance, hence, the problems associated with effective vector management. If insecticide resistance prevails in the face of the virus infection in mosquitoes, it would be difficult to control the vector and the arboviral infection would be sustained. The best approach to determining the level of infection rate in any endemic setting should consider the vectors rather than the blood samples of humans. This would inform policymakers on rollback actions against the vectors. A few studies in Africa have highlighted the susceptibility status of *Aedes* mosquitoes to some recommended insecticides and insecticidal materials [16,17].

One of the studies by Mukhtar MM *et al*. reported varying insecticide resistance with larvae and adults of *Aedes* [18]. Also, another study by Ojianwuna CC *et al*. equally reported the use of naphthalene combined with kerosene against *Aedes* larvae in Ika North East [19]. Another study by Ojianwuna CC *et al*. reported the effect of petroleum products on *Aedes* larvae in Ika North East [20]. The detection of YFV has relied on human subjects for decades. To confirm this, a study by Kolawole OM *et al*. reported the prevalence of arboviruses in individuals visiting health facilities in Nigeria [21]. This was done by molecularly screening arboviruses in blood samples of patients [22]. Sometimes in the absence of the detection kits, YFV infection may be misdiagnosed as malaria using related symptoms. Holistic intervention measures cannot rely on vaccines alone but the integration of intervention targeting the vectors, manipulation of their breeding environment, detecting the viral load in endemic species, and sequencing the viral genes to determine the virus responsible for the transmission in the location. Similarly, the acceptability and administration of vaccines for host protection is of utmost importance. The presence of the breeding sites of these vectors, as well as the infection in human subjects, show possibility of not only sustained infection in the future but also trans-ovarian transmission. As worrisome as this condition may be, not much has been done on detecting the strains of the virus in *Aedes* mosquitoes [23,24]. This study, therefore, determined the effectiveness of various sampling techniques and screened *Aedes* mosquitoes for the presence of YFV in Ika North LGA, Delta State.

## Methods

**Figure 1:**
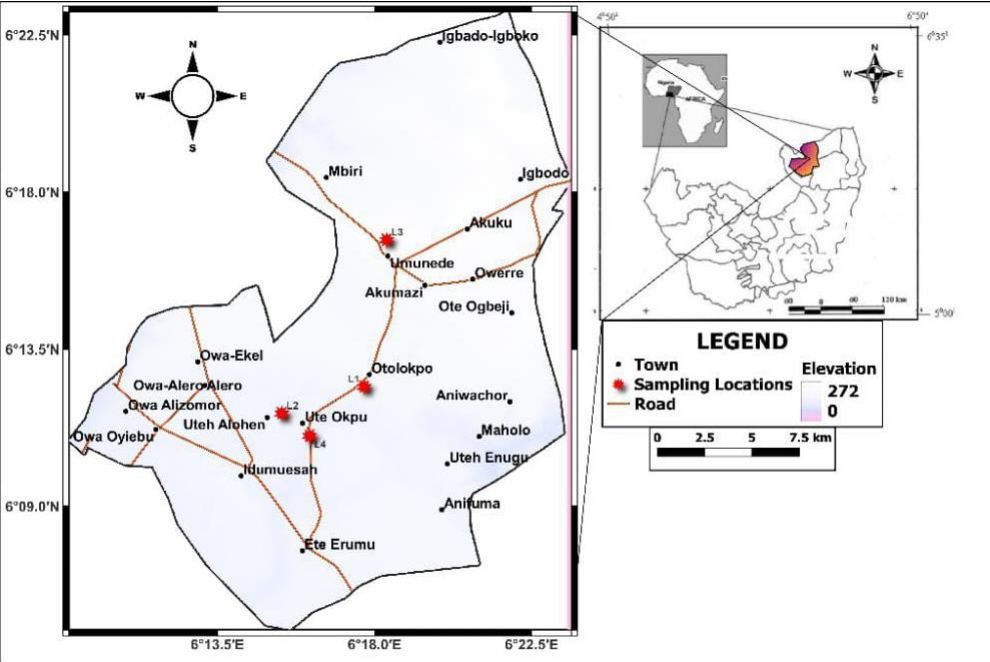
map showing sample locations in Ika North East LGA, Delta State, Nigeria

**Study area:** the field collection was done in Ika North East Local Government Area (LGA), Delta State, Nigeria. Four sample villages were selected for the study including Otolokpo (latitude: 6.100458 and longitude: 6.210345), Ute-Okpu (latitude: 6.094321 and longitude: 6.182408), Umunede (latitude: 6.154105 and longitude: 6.183491) and Ute Alohen (latitude: 6.121205 and longitude: 6.160588) (Figure 1). These sites were chosen due to the report and incidence of yellow fever and related arboviral cases in Ika North East [25].

**Materials:** the following materials were used in this study. They include: holding cages, aspirators, BG sentinel traps, CDC light traps, attractants, Eppendorf tubes, RNAi latter, and masking tapes.

**Study design:** this research work was designed to determine the efficacy of various sampling techniques of mosquitoes (odor-baited traps biogents sentinel (BG-sentinel), CDC light trap with attractant, CDC light trap without attractant, and modified human landing catch (m-HLC) in 12-weeks collection period). The human landing catch is a known traditional method of mosquito collection. It was suspended due to ethical issues surrounding the use of human subjects as bait. Given this, the landing catch using humans as bait was modified. More so, the study tried the efficacy of a CDC light trap baited with the synthetic lure. Mosquitoes were collected around houses associated with farms, blown into holding cages, killed by freezing, and immediately identified morphologically before pooling for preservation in Eppendorf tubes containing RNAi later. These mosquitoes were molecularly screened for YFV using YFV primers and probes. The Research and Ethics Committee of the Faculty of Science, Delta State University, Abraka, Nigeria approved this study with Ref No. REL/FOS/2023/03.

**Mosquito collection:** adult *Aedes* mosquitoes were collected from June to November 2021. The collection was done in rural communities of the LGA, where farming and different human impacts create potential breeding sites. Field collection was done using four sampling techniques for mosquito collection. The passive collection was done using BG-sentinel trap, a new trap designed to trap diurnal mosquitoes, *Aedes* to be specific, CDC light trap (CDC- LT), CDC light trap with pheromones (CDC-LT with Phe), while the active collection was done using modified human landing catch (m-HLC). These traps were set from 05: 30 am to 06: 00 pm and were positioned at strategic points where *Aedes* were perceived to be abundant. The collectors for the landing catch were vaccinated for yellow fever before the study. Modified human landing catches (m-HLC) were done using aspirators immediately after these mosquitoes perched on the clothing of the collector from 06: 00 am to 11: 30 am in the break of the morning and in the evening from 04: 00 pm to 07: 00 pm. The collectors wore thick black overalls covering the collector from head to toe except for the face. The clothing was rubbed with attractants containing L (+) - lactic acid, and hexanoic acid. This was done, in addition to the emission of carbon dioxide from the nose of the collector, to act as attractants for *Aedes* mosquitoes to the clothing. The collectors stood with shoulders side-by-side, one facing front to observe the back of the other and vice versa. The mosquitoes were transferred into the holding cage and killed by freezing at -4°C for 120 minutes.

**Environmental variables:** the temperature and relative humidity of the sampled points in the communities were taken using a thermohygrometer.

**Phenotypic identification, preservation, and molecular assay:** the mosquitoes collected from the field were phenotypically identified under a stereotyped dissecting microscope with the taxonomic guidelines by Farattini OP, to the genus level [26]. Further identification adopted the taxonomic guide by Cova-Garcia P *et al*. to species level [27]. These mosquitoes were then preserved immediately in Eppendorf tubes containing RNAi buffer, pooled in 15 mosquitoes per tube. Molecular speciation of *Aedes* mosquitoes was done using primers and probes of yellow fever in the Molecular Biology Laboratory, National Arbovirus and Vectors Research Centre, Enugu State, Nigeria.

**Deoxyribonucleic acid (DNA) extraction and amplification:** before extraction, the mosquitoes were pooled into eight reactions. The extraction was done using Zymo Research DNA extraction kit from the USA strictly following the user manual. The mosquitoes were identified phenotypically as *Aedes albopictus* and were only confirmed using the molecular approach. A total reaction volume of 20μl was adopted in the master mixing (reaction setup) for *Aedes albopictus*. This included solis Pre-mix (4μl), and reverse primer (Aeg & Alb) for *Ae. Albopictus* 0.6μl (5´- GTA CTA GGC TCA CTG CCA CTG A-3´), forward primer (18SFHIN) for *Ae. albopictus* 0.6μl (5´-GTA AGC TTC CTT TGT ACA CAC CGC CCT T-3´), nuclease-free water 11.8μl, and DNA template 3μl. For *Ae. aegypti* forward primer (5´ - TGGCTA GTC TGG ACGATGAAAGTGAC-3´) and reverser primer (3´GGTAGGTGGAATTTT GGGATGGTA GTC-5´).

One hundred (100) *Aedes* mosquitoes were randomly selected and molecularly identified by singleplex polymerase chain reaction (PCR) reaction method, using the protocol of Fanello C *et al*. [28]. The DNA of the mosquitoes was amplified in thirty-seven cycles. The first cycle was an initial denaturation done at 95°C for 5 minutes, another thirty-five cycles were done at 95°C, 55°C, and 72°C for 30 seconds respectively, and a final extension cycle was done at 72°C for 7 minutes. The amplicons were run on 2% agarose gel electrophoresis for a period of 1 hr 30 mins at 120 volts and the DNA bands were stained with ethidium bromide.

### Virus screening

**Ribonucleic acid (RNA) extraction:** before extraction, the mosquitoes were pooled into three reactions made up of fifteen mosquitoes each in a 2 ml Eppendorf tube. The extraction was done using Zymo Research RNA tissue/insect mini prep extraction kit from the USA strictly following the user manual.

**Cyclic DNA (cDNA) synthesis:** synthesis of the cyclic DNA was done using a cDNA synthesis kit from Bio Labs company, sourced from Inqaba Biotec West Africa. A total reaction volume of 20μl was used in the synthesis. This included template RNA 5μl, random primer 2μl, protoScript II Reaction (2X) 10μl, ProtoScript II Enzyme Mix (10X) 2μl, and Nuclease Free water 1μl. This synthesis was done by mixing the components in a PCR tube and incubating for 5 minutes at room temperature, transferring the mixture into an incubator at 42°C for 1 hour, and inactivating the enzyme at 80°C. Proceed with the synthesized cDNA for PCR amplification.

**Cyclic DNA (cDNA) amplification:** the molecular identification of YFV was done by singleplex PCR reaction, using the following amplification conditions. YFV reaction setup adopted another total reaction volume of 20μl including, Solis Pre-mix 4μl, YF1 0.6 μl (5´- AGA GTG AAA TTG TCA GCT TTG ACA CTC AAG GG-3´), YF2 0.6μl (5´- CCC TGA AAG GCA GAG CCA AAC ACC-3), nuclease free water 11.8μl, and cDNA template 3μl. The method of Bagnoli JW *et al*. was adopted [29]. The amplification was done in thirty-seven cycles including an initial denaturation which was done in one cycle at 95°C for 4 minutes, another thirty-five cycles done at 95°C and 50°C for 30 seconds respectively, and 72°C for 60 seconds, and a final extension cycle done at 72°C for 8 minutes. The amplicons were run on 2% agarose gel electrophoresis for a period of 1 hr 30 mins at 120 volts and the DNA bands were stained with ethidium bromide.

**Statistical analysis:** the mean abundance of *Aedes* mosquitoes from the various sampled points in each community was entered into an Excel spreadsheet and double-checked before analysis. The mean abundance of *Aedes* mosquitoes in the sampled communities was subjected to the Analysis of Variance (ANOVA) test to ascertain the level of significance p=0.05. Canonical correspondence analysis (CCA) was used to check for the relationship between *Aedes* mosquito abundance and environmental variables. Abundance was predicted for the area using the collection techniques. All molecular analysis was done using Biorad CFX Manager and analyzed using CFX Manager Software Version 3.1.3086.0516, 2018 Bio-Rad Laboratories, Inc.

**Ethical consideration:** the Research and Ethics Committee of the Faculty of Science, Delta State University, Abraka, Nigeria approved this study with Ref No. REL/FOS/2023/03.

## Results

**Figure 2:**
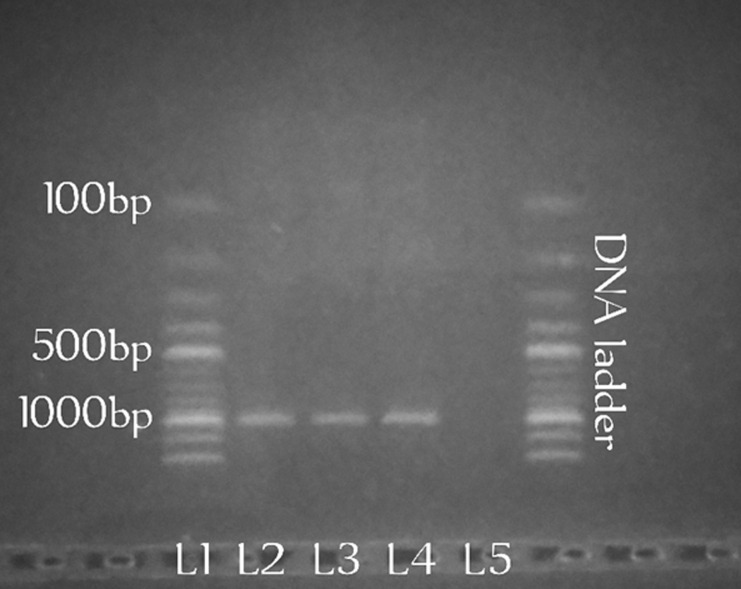
electrophoresis chart for *Aedes albopictus*

**Sampling efficiency:** seven hundred and twenty-five (725) mosquitoes were encountered in this study. The one hundred *Aedes* mosquitoes randomly selected for molecular identification were confirmed as *Aedes albopictus* (Figure 2). The modified human landing catch techniques recorded the highest mean mosquito abundance (42.86). The difference between m-HLC and other methods was significant (p< 0.05). The mean abundance of *Aedes* mosquitoes was lowest with the CDC trap (0.29) (Table 1).

**Table 1:** mean abundance of *Aedes* mosquitoes with various sampling techniques in selected communities in Ika North East, Delta State

Sampling methods	Mean (n)	Standard error	The lower bound (95%)	Upper bound (95%)	Tukey test	
CDC trap	0.29 (4)	2.82	-5.378	5.950	A	
CDC trap + attractant	3.50 (49)	2.82	-2.164	9.164	A	
BG-Sentinel trap	5.14 (72)	2.82	-0.521	10.807	A	
m-HLC	42.86 (600)	2.82	37.193	48.521		B

**Note:** group means with similar letters do not differ significantly (p>0.05); CDC: Centers for Disease Control; BG: biogents; m-HLC: modified human landing catch

**Figure 3:**
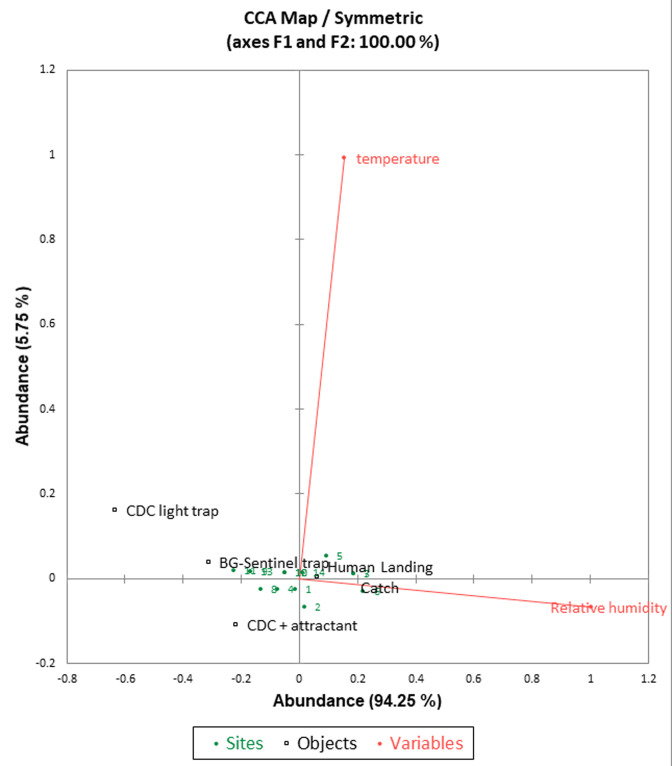
canonical correspondence analysis (CCA) plot of *Aedes* mosquitoes with temperature and relative humidity in Ika North East LGA, Delta State, Nigeria

**Canonical correspondence biplot:** the permutation test concludes that mosquito abundance is not linearly related to the environmental variables (p=0.376). Canonical correspondence analysis of *Aedes* mosquitoes with temperature and relative humidity in Ika North East LGA (Figure 3), shows that the efficiency of capturing *Aedes* mosquitoes using the m-HLC is associated with high humidity and low temperature. More so, the efficiency of capture using the traps is associated with low temperature and humidity.

**Figure 4:**
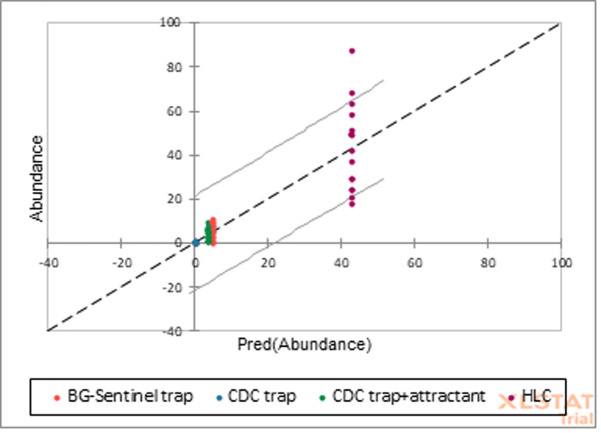
prediction model of *Aedes* mosquito abundance using the different sampling techniques in Ika North East, Delta State, Nigeria

**Prediction model for *Aedes* abundance:** the prediction model of *Aedes* mosquito abundance using the different sampling techniques in Ika North East (Figure 4) shows that m-HLC would catch 87% *Aedes* mosquitoes out of 100% in the field while others such as BG sentinel trap, CDC trap with attractant, and CDC trap alone would catch 11%, 9% and 1% of *Aedes* mosquito species.

**Figure 5:**
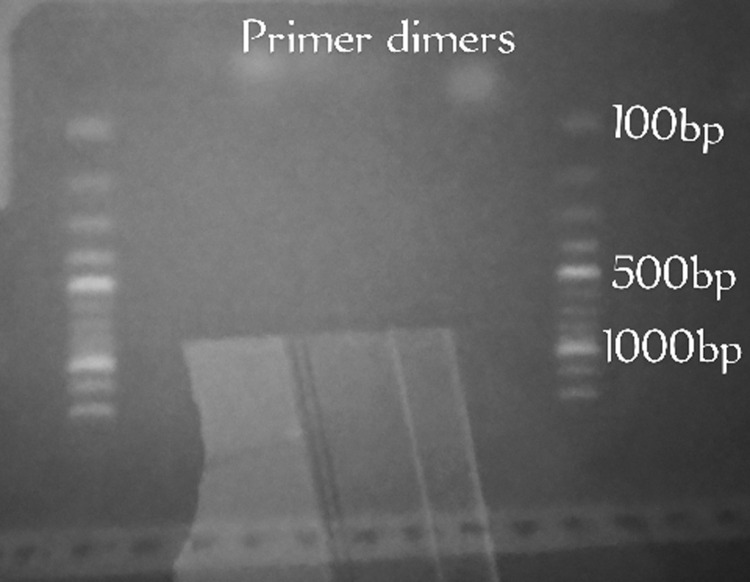
electrophoresis chart for yellow fever analysis

**Yellow fever virus quantification:** the sampled mosquitoes tested for yellow fever virus strain showed no virus in the four locations, which is inferred as the absence of a band expected to be 486bp (Figure 5). This could be due to the absence of a viral genome in the mosquitoes collected. This could equally be that the viral coverage for the mosquitoes to pick up in blood meal has been suppressed by the mass vaccination in the area.

## Discussion

In the current study, *Aedes* mosquitoes were sampled using odor-baited traps (BG-sentinel), CDC light traps with attractant, CDC light traps without attractant, and m-HLC in 12-weeks collection period and resulted in seven hundred and twenty-five *Aedes* mosquitoes from four locations in Delta State, Nigeria. The number of *Aedes* mosquitoes collected from this study corresponded to the study of another study where over seven hundred mosquitoes were collected from Southwestern Nigeria [30]. However, they reared their larvae to adults before counting while in this study adults were collected using various techniques to ascertain efficiency.

The findings of this present study are inconsistent with the previous study reported by Cansado-Utrilla C *et al*. that recorded over ten thousand *Anopheles* mosquitoes using Stealth traps, CDC light traps, gravid traps, and BG-Sentinel traps [31]. Similarly, a five-month Ethiopian study by Kenea *et al*. (2017) recorded over seven thousand *Anopheles* mosquitoes. Some studies have tested the efficacy of existing and novel traps in capturing *Anopheles* and *Culex* mosquitoes (Mwanga *et al*. 2019; Cansada-Utrilla *et al*. 2020; Sanou *et al*. 2021), these equally have been compared with human landing catches [32]. A Southeastern Nigeria study by Egwu O *et al*. [33], revealed that the abundance of *Aedes* mosquitoes collected and reared from their breeding habitat was below five hundred which is lower than those recorded in this study. More so, the mosquitoes molecularly tested in this study were all *Aedes albopictus*. Due to the paucity of information on capturing *Aedes* mosquitoes, this study was designed to ascertain whether the sampling methods proposed in this study would be valuable in capturing the yellow fever vectors. Thus, the low number of *Aedes* species in this study could be attributed to the fact that the species targeted in the previous studies were different, and also, the sampling duration could have influenced a large number of species by the study reported [31,34,35]. The m-HLC technique recorded the highest mean mosquito abundance than the other sampling techniques in this study. The study of Maliti DV *et al*. [36], revealed that human landing catches compared favorably to the electrocuting traps for mosquitoes outdoors and indoors. However, the commercial electrocuting trap in their study performed poorly. None of the traps in this study corresponded to m-HLC catches. CDC light traps performed poorly in the collection of *Aedes* mosquitoes but were at best compensating when enhanced with *Aedes* sex hormone attractants.

Human landing catch is a traditionally known golden standard still in use today for the collection of mosquitoes [37]. The method is an active, labor, and ethically demanding activity and could have been the reason why more species are always collected compared to the passive method [37]. In this present study modified human landing catch was adopted to reduce to a minimum the chances of mosquito biting activities on collectors. This probably explained why m-HLC in this study got the highest species compared to the enhanced and normal passive method for *Aedes* mosquito collection. The use of attractant-enhanced traps equally gave high mosquito species. Their inability to produce many results in terms of species abundance may be due to the absence of carbon dioxide common with human productions. Although the use of traps in other locations in West Africa reported high catches including in Guinea [38], Sierra Leone [39] and gravid traps in Ghana [40], especially with BG-sentinel traps. Some authors have argued that the efficiency of various traps may be species-specific. For instance, Opoku M *et al*. [41], ascribed the high abundance of *Anopheles* mosquito caught by CDC light traps to the intensity of light which shows that they are designed for night-biting species. The CDC light may probably not have an impact on day-biting species like *Aedes*. BG-sentinel trap was created for the collection of *Aedes* and has been used with success in Burkina Faso [42]. A study has confirmed that BG-sentinel traps, in general, are effective for catching *Aedes* mosquitoes and that the inclusion of attractants improves the collection [43]. To check whether the attractant is responsible for the catches, the CDC light trap was enhanced with a similar attractant. There was a slight increase in mosquitoes in the CDC-enhanced trap to the normal CDC trap. The study of Wilke ABB *et al*. [44], confirmed that the addition of an attractor to the BG trap increased the efficiency of the capture of *Aedes* in Florida.

In sampling mosquitoes for disease determination, *Aedes* mosquitoes cannot be neglected in the fight against mosquito-borne diseases since they are known to cause numerous arthropod viruses in humans and animals alike in Africa [6,45]. However, reports by Estrada-Franco JG *et al*. [46], have shown that *Culex* especially the *pipiens* and *quinquefasciatus* species complexes and *Anopheles* equally transmit arboviruses in some locations. *Aedes* mosquitoes are abundant and distributed in Nigeria due to their ability to explore potential habitats associated with humans and farm settlements [1]. Apart from the fact that yellow fever is prevalent in Nigeria, *Aedes* have been reported to cause Dengue fever virus and chikungunya infections [7,47]. World Health Organization (WHO) in 2020, found that a strange illness spread through some communities of Ika North East including Otolokpo, Ute-Okpu, Idumesah and some as danger villages, and was confirmed as yellow fever following several blood tests [25]. To date, no study has been carried out to detect the yellow fever virus in the drivers of this deadly disease. However, vector control through the innovative application of petroleum products in breeding sites has been reported [20]. More so, studies by Ojianwuna CC *et al*. and Guarido MM *et al*. have tried to monitor the emergence inhibition of *Aedes* mosquitoes to naphthalene and kerosene mixtures, and pyrethroid insecticide [19,48]. The detection of yellow fever and other arboviral infections in blood samples of humans is a common practice in Nigeria. However, research attention has been drawn not only to detect viral agents in blood but to screen mosquito vectors for possibilities of viral strains. The arboviral infection has been in occurrence since the mid-nineteenth century till its spread caused global challenges [45]. Detection of arboviruses in mosquitoes is a recent research adventure in Nigeria. There is a paucity of information in the literature on Delta State. This study, therefore, stands out as a baseline inquiry on the efficiency of various sampling techniques and screening of the yellow fever virus strain from *Aedes* mosquitoes in Ika North East, Delta State, Nigeria. This study was designed due to the report of a yellow fever outbreak in Delta State in the last three years [25].

Permutation test of canonical correspondence analysis between the *Aedes* mosquitoes and environmental variables showed that mosquito abundance was not linearly related to the environmental variables. Guarido MM *et al*. revealed that temperature was the major factor influencing the presence of *Aedes* mosquitoes [49]. It was observed in this study that the *Aedes* mosquito captured using the m-HLC is associated with high humidity and low temperature. More so, the efficiency of capture using the traps is associated with low temperature and humidity. Moisture and temperature are a function of the current status of the environment. In forested areas, like that of Ika North East where palm plantations are prolific as well as other trees, the likelihood of moisture deficiency especially in the early hours of the morning and late is low. The prediction model showed that modified human landing catch could catch 87% of *Aedes* mosquitoes while others such as BG-sentinel trap, CDC trap with attractant, and CDC trap alone could catch 11%, 9%, and 1% of *Aedes* mosquito species. No yellow fever strain was encountered in screened mosquitoes with the amplified cycles in this present study. This could be ascribed to that the vaccination conferred protection on the residents and thus reduced the chances of yellow fever virus strain in *Aedes* and that the chances of trans-ovarian transmission are very low in these mosquitoes. This result did not correspond to the study of Agwu EJ *et al*. which detected yellow fever and Dengue viruses in *Aedes* mosquitoes from *Ae. autocephalous, Ae. aegypti* and *Anopheles gambiae* in Bayelsa and Benue [50]. Similarly, Mutebi JP *et al*. revealed a 100% infection rate, 20% dissemination rate, and the transmission of CENETROP-322 of the Bolivian YFV strain in their study [51]. Pinheiro GG *et al*. equally reported an infectivity rate of 8.2% in Minas Gerais State, Brazil [52].

## Conclusion

This study has shown that the modified human landing catch (m-HLC) and to some reasonable extent BG-sentinel trap are best at trapping *Aedes* mosquitoes in endemic communities in this area. *Aedes albopictus* was the dominant vector and was observed not to have the viral strain. This predicts that the vaccination strategies previously carried out suppressed infection in the population. However, this does not imply that there is no likelihood of yellow fever in the human population. Thus, more attention is required to redetect the mosquitoes constantly for possibilities of viral reinfection and careful monitoring is advised.

### What is known about this topic

- *Aedes mosquitoes are generally known vectors of the yellow fever virus among other arthropod-borne viruses;*
- *Aedes mosquitoes are day-biting species with a wide range of breeding habitats*.

### What this study adds

- *Aedes mosquitoes can be effectively trapped using modified human-baited catches;*
- *Aedes mosquitoes in this location were typically all Ae. albopictus;*
- *Yellow fever virus was not detected in the species of mosquitoes which predicts that mass vaccination could have reduced viral strains in the human population*.
